# Discrete Element Model for Suppression of Coffee-Ring Effect

**DOI:** 10.1038/srep42817

**Published:** 2017-02-20

**Authors:** Ting Xu, Miu Ling Lam, Ting-Hsuan Chen

**Affiliations:** 1 Department of Mechanical and Biomedical Engineering, City University of Hong Kong, 83 Tat Chee Avenue, Hong Kong Special Administrative Region, China; 2 School of Creative Media, City University of Hong Kong, 83 Tat Chee Avenue, Hong Kong Special Administrative Region, China; 3 Centre for Robotics and Automation, City University of Hong Kong, 83 Tat Chee Avenue, Hong Kong Special Administrative Region, China; 4 CityU Shenzhen Research Institute, 8 Yuexing 1st Road, Shenzhen Hi-Tech Industrial Park, Nanshan District, Shenzhen 518057, China

## Abstract

When a sessile droplet evaporates, coffee-ring effect drives the suspended particulate matters to the droplet edge, eventually forming a ring-shaped deposition. Because it causes a non-uniform distribution of solid contents, which is undesired in many applications, attempts have been made to eliminate the coffee-ring effect. Recent reports indicated that the coffee-ring effect can be suppressed by a mixture of spherical and non-spherical particles with enhanced particle-particle interaction at air-water interface. However, a model to comprehend the inter-particulate activities has been lacking. Here, we report a discrete element model (particle system) to investigate the phenomenon. The modeled dynamics included particle traveling following the capillary flow with Brownian motion, and its resultant 3D hexagonal close packing of particles along the contact line. For particles being adsorbed by air-water interface, we modeled cluster growth, cluster deformation, and cluster combination. We found that the suppression of coffee-ring effect does not require a circulatory flow driven by an inward Marangoni flow at air-water interface. Instead, the number of new cluster formation, which can be enhanced by increasing the ratio of non-spherical particles and the overall number of microspheres, is more dominant in the suppression process. Together, this model provides a useful platform elucidating insights for suppressing coffee-ring effect for practical applications in the future.

When a sessile droplet rests on a horizontal surface, the water content gradually evaporates and leaves a ring-shaped stain after drying, which is called the “coffee-ring effect”^1^,^2^,^3^. This phenomenon is due to an enhanced evaporation at the droplet edge while the contact line remains pinned^1^,^2^,^3^,^4^. Hence, to compensate the increased evaporative loss at the droplet edge, a capillary flow from the interior to the contact line is generated, which moves the suspended particulate matters towards the droplet edge, finally forming a ring-shaped deposition. Apparently, due to the consequence of coffee-ring effect, it is undesired in many applications, such as inkjet printing^5^ or protein microarrays^6^ that requires uniform coating of solid contents after drying^1^,^2^,^3^,^4^,^7^,^8^. As such, intensive efforts have been made to eliminate the coffee-ring effect, including capillary force^9^, Marangoni effect^8^, and addition of surfactant^10^.

Recently, Yunker and co-workers found that suspended ellipsoids would suppress the coffee-ring effect and obtain a uniform deposition of particles^4^. It is because the micrometer-sized particles with large aspect ratios, e.g. ellipsoids^4^ and rod-like particles^11^, tend to cling to and float at the air-water interface. Because the air-water interface surrounding the floating non-spherical particles is greatly distorted, it would cause a significantly enhanced particle-particle attraction^11^,^12^, leading to a formation of monolayer network of particles with increased fluidic resistance that prevents the particles from being carried to the droplet edge. More importantly, when mixing with microspheres, a small amount of ellipsoids is sufficient to destroy the coffee-ring effect^4^. This phenomenon was recently developed into a bio-sensing mechanism, in which coffee-ring effect was suppressed based on the generation of non-spherical particle agglomerates when target molecules were present^13^.

To comprehend the physics underlying the coffee-ring effect, numerical simulation including Monte Carlo model^14^,^15^,^16^ and Finite Element Method^7^,^17^ has been sought. However, they are both limited. Finite Element Method is based on continuum mechanics, which makes it difficult to simulate the particle-particle interactions in discrete forms. In the Monte Carlo model, as it uses probability distribution to manipulate the particles with biased random walk, the model can only simulate simplified particle motions in a fixed lattice without accurately reflecting the influence of hydrodynamics and particle-particle interaction. Here, we report a discrete element model (particle system) to investigate the suppression of coffee-ring effect based on the interactions of spherical and non-spherical particles. The particles in the bulk solution followed the capillary flow (Fig. 1a), and became 3D hexagonal close packing when reaching the substrate or along the contact line. To simulate the particle-particle interaction when they were adsorbed by air-water interface, we implemented cluster growth, cluster deformation, and cluster combination. We found that the coffee-ring effect can be effectively suppressed when the flow in the bulk solution and at air-water interface were both outward, which is different from the previous notion that a circulatory flow with an inward flow at air-water interface is required. In contrast, we found that the suppression of coffee-ring effect is highly dependent on the number of new cluster formation, which can be enhanced by increasing the ratio of non-spherical particles and the overall number of microspheres. Taking together, this discrete element model provides a valuable tool for studying the suppression of coffee-ring effect in the future.

## Results

**Figure 1:**
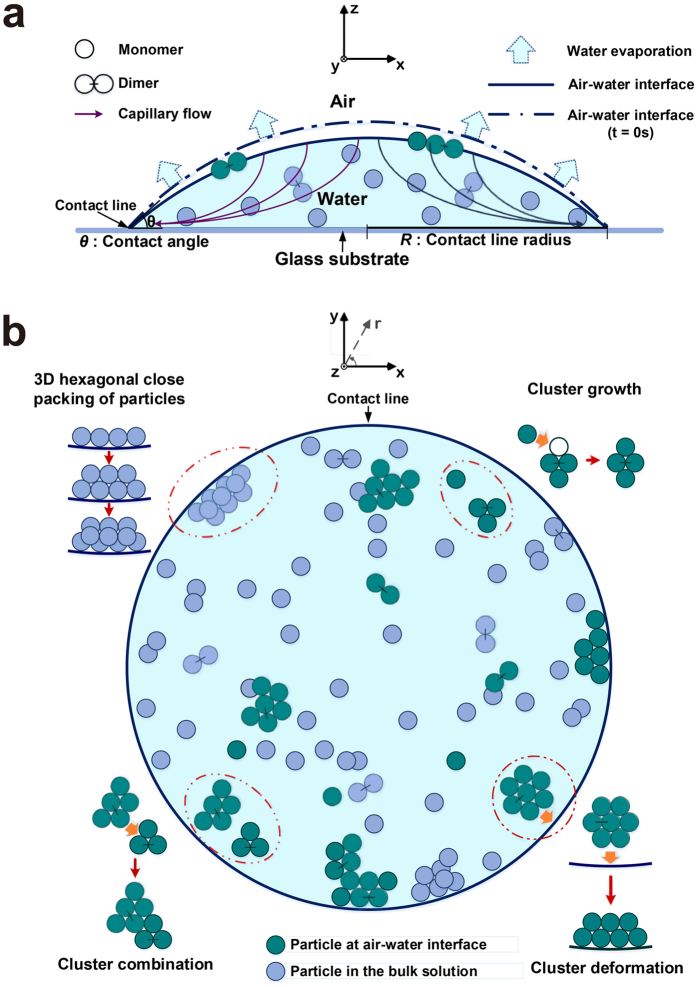
The schematic diagram of a water droplet evaporating on a flat substrate. (**a**) The side view showing that the outward capillary flow carries suspended spherical monomers (single microspheres) and non-spherical dimers (pairs of monomers) to the droplet edge during evaporation. While the contact line is pinned, the droplet height is descending due to the evaporation. (**b**) The top view showing the simulated particle-particle interactions as 3D hexagonal close packing of particles in the bulk solution (upper left), cluster growth at air-water interface (upper right), cluster combination at air-water interface (lower left), and cluster deformation at air-water interface (lower right).

A continuous 3D domain was shaped as a spherical cap mimicking a pinned sessile droplet containing two types of suspended particles, monomers (single microsphere with diameter *d* = 1 μm) and dimers (a pair of monomers) (Fig. 1a). During evaporation, the height of domain boundary decayed and adsorbed the particles to the air-water interface. The particle movement in the bulk solution was modeled using the analytical solution of the capillary flow with Brownian motion (Fig. 1a, see Supplementary Information). When the particles were near the substrate or droplet edge, they were assembled with 3D hexagonal close packing (Fig. 1b, the upper left corner). Once a dimer was adsorbed by air-water interface, it initiated a cluster growth as a particle monolayer with hexagonal close packing by recruiting other monomers at the air-water interface to become part of the cluster (Fig. 1b, the upper right corner). After the cluster formed, while moving following the surface fluid flow as a whole, each cluster was allowed to combine with others through a stochastic approach (Fig. 1b, the lower left corner). Furthermore, when approaching to the region close to the droplet edge, large cluster stopped and became immobilized, whereas small cluster would deform and be reorganized into a particle monolayer with hexagonal close packing above the 3D ring structure (Fig. 1b, lower right corner).

**Figure 2:**
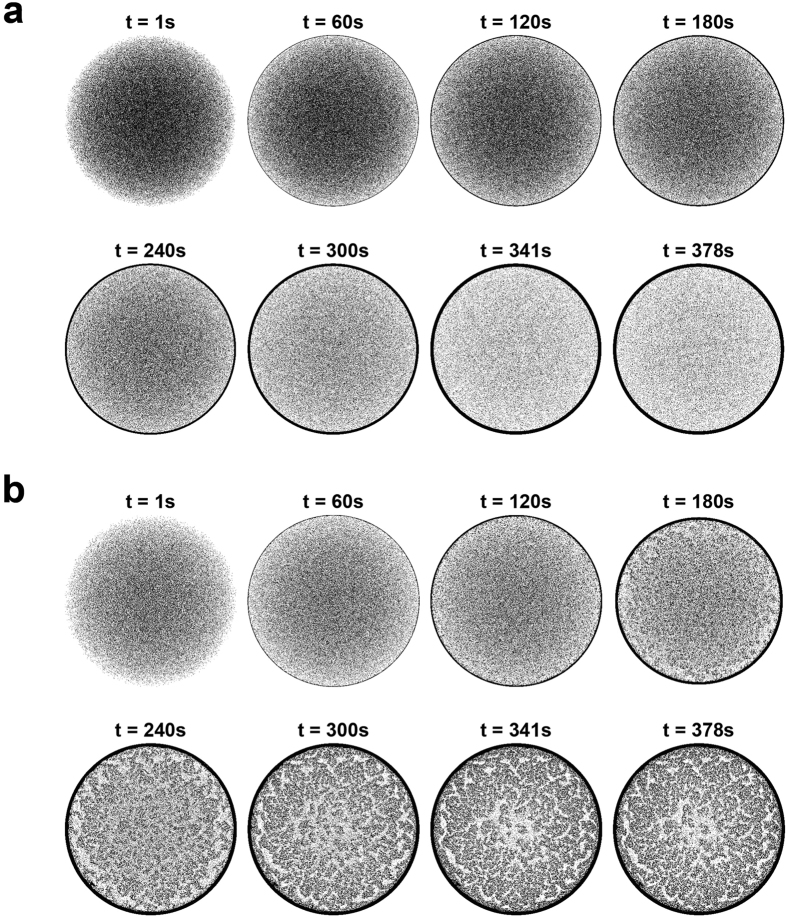
Dynamics of coffee-ring formation. (**a**) A ring-shaped deposition formed by 1,500,000 microspheres with 100% monomers. (**b**) A uniform deposition of 1,500,000 microspheres with 100% dimers.

We first studied the dynamics of coffee-ring suppression using only spherical monomers or non-spherical dimers. Initially, particles were uniformly suspended in the continuous 3D domain, and more particles were observed in the center due to dome shape of the sessile droplet. During evaporation, when there were only monomers within the droplet, the outward capillary flow carried particles all the way toward droplet edge, leading to a formation of a ring-shaped deposition (Fig. 2a). As the evaporation process approached to the end, the contact line started to recede, which forced the suspended particles to directly sink onto the substrate. Eventually, the uniformly distributed particles in the initial condition became highly concentrated along the droplet edge. In contrast, when there were only dimers, the non-spherical dimers might move to the droplet edge or being adsorbed at the air-water interface during evaporation (Fig. 2b). Subsequently, adsorbed the dimer would initiate the cluster growth and cluster combination, forming a monolayer network of particles at the air-water interface that suppressed the coffee-ring effect.

**Figure 3:**
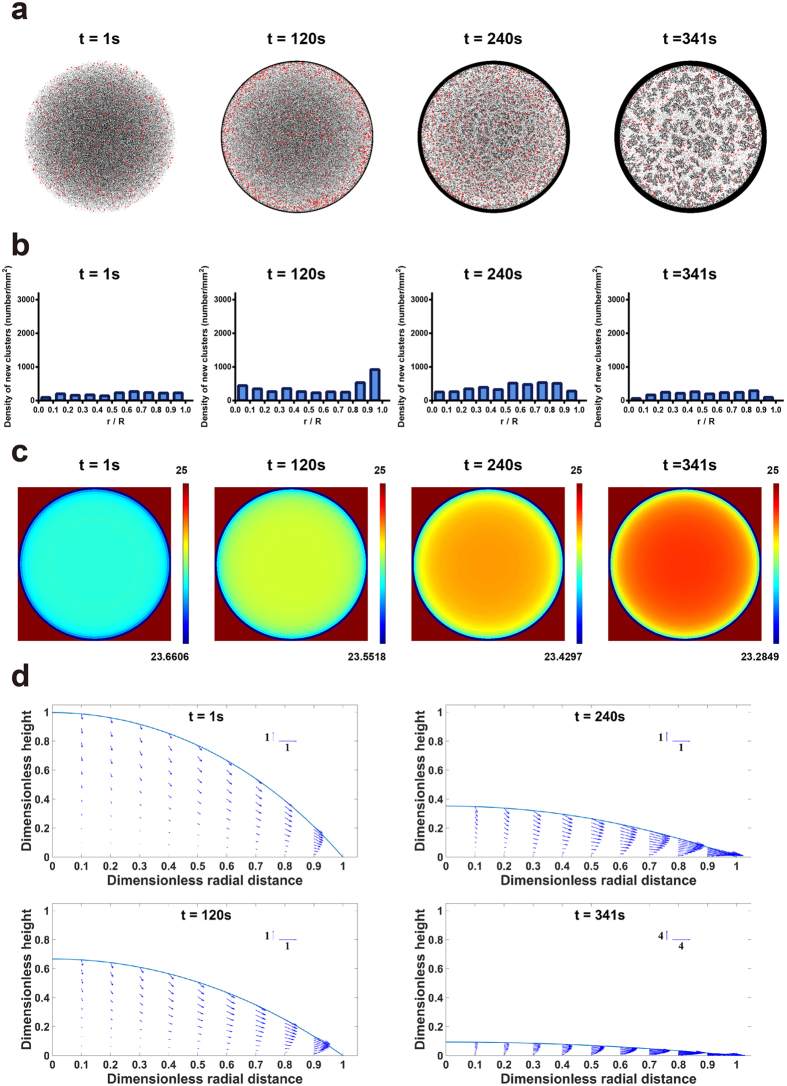
Formation of new clusters depending on the outward capillary flow. (**a**) The top view of newly adsorbed dimers (labeled red) at different time based on total 1,500,000 microspheres with 66.7% dimers (*w/w*). (**b**) The density distribution of newly adsorbed dimers during evaporation based on total 1,500,000 microspheres with 66.7% dimers (*w/w*). (**c**) The color map of temperature distribution during evaporation. (**d**) The velocity field of outward flow at the side view.

Apparently, the suppression of coffee-ring effect is dependent on the adsorption of dimers that initiated the particle-particle interactions at air-water interface. We next studied the distribution of newly adsorbed dimers at different time. By analyzing the density distribution of newly adsorbed dimers, we can observe that dimers were adsorbed uniformly in most of time (Fig. 3a,b). However, there was a slight increase of adsorbed dimers near the droplet edge when *t* = 120 s (Fig. 3b), suggesting an influence of the outward flow. To illustrate the outward flow, we investigated the flow field and temperature distribution over time. Remarkably, during the evaporation process, the enhanced evaporation near the contact line resulted in a lower temperature at droplet edge (Fig. 3c), which caused an outward Marangoni flow at air-water interface based on the Marangoni effect^8^. As a result, the flow in the bulk solution and at air-water interface were both outward, and no circulatory flow was observed (Fig. 3d). Notably, the coffee-ring effect can still be suppressed with such outward flow, suggesting that the circulatory flow driven by an inward surface flow may not be required.

**Figure 4:**
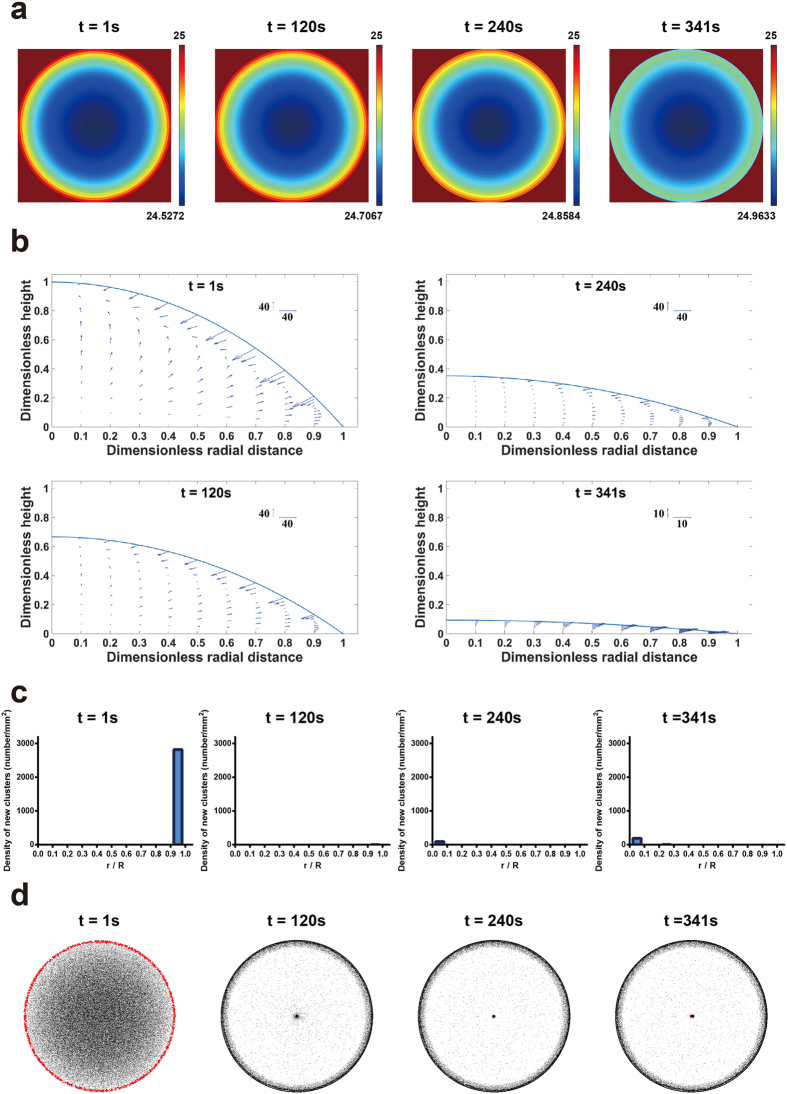
Reversed surface flow induced by Marangoni effect with increased thermal conductivity of the substrate (*k*_2_ = 100 W/m·K) and the Marangoni number (Ma = 1000). (**a**) The color map of temperature distribution during evaporation. (**b**) The velocity field of the circulatory flow at the side view. (**c**) The histogram of the location of newly adsorbed dimers during evaporation based on total 1,500,000 microspheres with 66.7% dimers (*w/w*). (**d**) The top view of newly adsorbed dimers (labeled red) at different time based on total 1,500,000 microspheres with 66.7% dimers (*w/w*).

We next applied our model to further investigate the role of the circulatory flow. To implement this, the thermal conductivity of substrate (*k*_2_) was adjusted from 1.1 to 100 W/m·K, by which the temperature distribution was reversed, i.e. lower temperature at the droplet center (Fig. 4a). Also, the Marangoni number (Ma) was increased from 8 to 1000 to yield a stronger Marangoni effect. As a result, while the bulk flow was still outward, the Marangoni effect reversed the surface flow at air-water interface from outward to inward and generated a circulatory flow as seen from the side view (Fig. 4b). Surprisingly, the newly adsorbed dimers was only observed near the droplet edge when *t* = 1 s, and the overall number of newly adsorbed dimers decreased (Fig. 4c). The flow field suggests that a very strong outward flow in bulk solution was created and carried many particles to the edge (~40 times faster than that in Fig. 3d, see scale bars), which may explain the increased adsorption of dimers when *t* = 1. Also, a downward flow near the air-water interface was also created, which prevented the adsorption of dimers and the subsequent cluster formation in the inner region (Fig. 4b). As a result, while the circulatory flow was created, the suppression of coffee-ring effect was even less (Fig. 4d). Moreover, by keeping *k*_2_ = 100 W/m·K and changing the Ma back to 8, which is an approximation by fitting the experimental measurement of a water droplet^8^, we found that the temperature distribution was still reversed but the circulatory flow disappeared (Supplementary Fig. S1a,b). As a result, the distribution of newly adsorbed dimers and coffee-ring formation were similar to that of regular settings (Supplementary Fig. S1c,d and Fig. 3). Consequently, for the suppression of coffee-ring effect based on non-spherical particles at air-water interface, the circulatory flow caused by the Marangoni effect is unnecessary and should be avoided.

**Figure 5:**
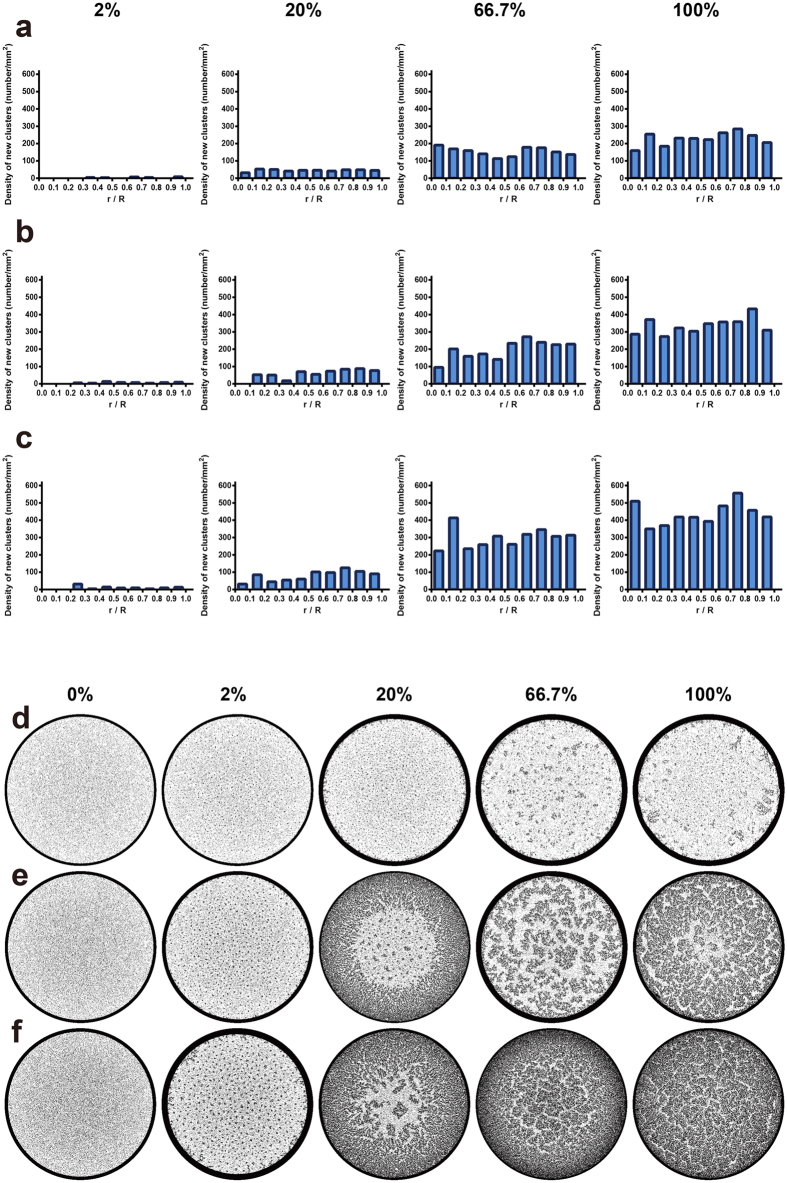
Suppression of coffee-ring effect based on mixed dimers and monomers. (**a–c**) The histogram of the location of newly formed clusters with total 1,000,000 (**a**), 1,500,000 (**b**), or 2,000,000 (**c**) microspheres containing different percentage of dimers (*t* = 1 s). (**d–f**) The simulated coffee-ring pattern based on total 1,000,000 (**d**), 1,500,000 (**e**), or 2,000,000 (**f**) microspheres containing different percentage of dimers.

As the suppression of coffee-ring effect is associated with the enhanced particle-particle interactions and the corresponding network formation at air-water interface, the number of adsorbed dimers and monomers should be essential. We next studied the distribution of newly adsorbed dimers in response to the ratio of non-spherical particles and the total number of microspheres. Using mixed monomers and dimers with different ratios (0%, 2%, 20%, 66.7%, and 100%, *w/w*, total 1,000,000 microspheres), we found that the number of newly adsorbed dimers can be effectively increased when the ratio of non-spherical particles increased (Fig. 5a). Such increase was further enhanced when the total number of microspheres was increased to 1,500,000 or 2,000,000 (Fig. 5b,c). Moreover, the simulated deposition pattern showed that the coffee ring was not suppressed using total 1,000,000 microspheres (Fig. 5d). In contrast, effective suppression was observed by cases of 1,500,000 or 2,000,000 microspheres (20–100%, Fig. 5e,f). These results are consistent with our previous findings that the suppression of coffee-ring effect can be enhanced by increased number of non-spherical particle agglomerates when more target molecules were present^13^. Overall, we demonstrated the number of newly adsorbed dimers plays a significant role, and it can be enhanced by increasing the ratio of non-spherical dimers and the overall number of microspheres.

## Discussion

In this study, we report a discrete element model (particle system) to simulate the suppression of coffee-ring effect. Based on the pinning effect of the contact line and the enhanced evaporation at the droplet edge, a self-driven capillary flow constantly pushed suspended particles from the interior to the edge. Meanwhile, due to the distorted air-water interface surrounding the floating non-spherical dimers, dimers would be adsorbed by the descending air-water interface and recruited other monomers to initiate the cluster formation^4^,^13^. As a result, if only monomers were present, particles were concentrated to the droplet edge and formed a ring pattern at the end. In contrast, if dimers were present, clusters were formed at air-water interface. Subsequently, combining with the cluster growth, combination, and deformation, the particle-particle connection was iteratively increased, eventually forming a macroscopic network that suppressed the coffee-ring effect.

Notably, we found that the coffee-ring effect can be suppressed when the surface flow was outward. This is different from the previous notion in which coffee-ring suppression needs an inward surface flow for the generation of a circulatory flow^14^. In this particle system model, with the absence of circulatory flow, the particle-particle interaction could still establish a network structure at air-water interface that amplified the fluidic resistance and prevented particles from being carried to the droplet edge. As such, the inward surface flow is not required, and should be even avoided since the circulatory flow based on Marangoni effect would introduce an additional downward flow near the air-water interface, reducing the adsorption of dimers. Moreover, as the generation of an inward surface flow needs strong Marangoni effect, which can’t be easily achieved in water-based droplet, the unnecessity of the inward surface flow suggests a wider practicability of this mechanism.

Moreover, we also found that the coffee-ring effect can be more effectively suppressed by increasing the number of newly adsorbed dimers, which can be enhanced by increasing the ratio of non-spherical particles or the overall number of microspheres. Interestingly, for total 2,000,000 microspheres, the coffee-ring effect was effectively suppressed in low concentration of dimers (20%), in which the number of newly adsorbed dimers was even lower than the case of 1,000,000 or 1,500,000 microspheres with higher ratio of non-spherical particles (66.7–100%). This result suggests that with high number of microspheres, monomers at air-water interface can be quickly recruited by other adsorbed dimers, resulting in a faster growing and larger size of clusters that eventually interconnect into a network covering the entire air-water interface.

In summary, this model incorporates the analytical solution of droplet evaporation and its resultant capillary flow. Based on this continuous 3D domain with decaying dome-shaped air-water interface, we achieved a first discrete element model incorporating a variety of particle motions, including particle movement following capillary flow with Brownian motion, formation of 3D hexagonal close packing in the bulk solution, cluster growth and cluster combination at air-water interface, and cluster deformation when encountering the droplet edge. Together, this simulation model provides a valuable tool for achieving an effective suppression of coffee-ring effect for practical applications in the future.

## Method

### Mathematical Model

This computational simulation is based on a discrete element model (particle system) in a continuous 3D domain. The 3D domain was shaped as a spherical cap mimicking a pinned sessile droplet evaporating on a flat substrate. The droplet is 0.6 μl in size, with an initial contact angle as 40° (Fig. 1a). The assumption of spherical cap is valid for small value of Bond number (Bo) and capillary number (Ca) (see Supplementary Information for details)^7^. The domain contains two types of suspended particles, monomers (single microsphere with diameter *d* = 1 μm) and dimers (a pair of monomers), and the total number of microspheres is 1,000,000, 1,500,000 or 2,000,000 (a dimer counts two microspheres). In this setting, the highest particle volume fraction is 0.0018 (2,000,000 microspheres in 0.6 μl droplet), which is low such that the presence of particles would not change the viscosity of water or transform the water from Newtonian fluid to non-Newtonian fluid^18^.

Two evaporation phases were conducted. In phase 1, the contact line was pinned while the droplet height, *h*, continuously decayed in the vertical direction^3^,^7^,^19^. The *h* and the contact angle *θ* were updated every second based on the approximate evaporation rate, 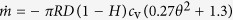
7, in which *R* is the contact line radius, *D* is the vapor diffusivity, *c*_v_ is the saturated vapor concentration on the droplet surface, and *H* indicates the relative humidity of the ambient air. When *θ* became 4°, the phase 2 started, during which *θ* was kept at 4° while the contact line gradually receded^7^. Afterward, the evaporation was completed when the contact line radius shrunk to zero. The total duration of both phase 1 and 2 was then denoted as the total drying time *t*_f_.

In phase 1, we modeled multiple particle motions, including the movement of suspended particles following capillary flow with Brownian motion, the formation of 3D hexagonal close packing of particles in the bulk solution, and cluster growth, cluster combination, and cluster deformation at air-water interface. Same with the shape change of the 3D domain, the position of particles was updated every second (*t*_next_ − *t*_now_ = 1 s). A systematic framework was adopted to determine the status of each particle so an appropriate particle motion can be applied (Supplementary Fig. S2). In phase 2, as the droplet height, *h*, was reduced to nearly zero, particles continuously descended until they touched the substrate. The entire process is summarized in a flow chart (Supplementary Fig. S2) and described in details in follows.

In phase 1, the particles were all suspended in the bulk solution at beginning, (*Status* = *Bulk*, Supplementary Fig. S2). The suspended particles first moved following the capillary flow with Brownian motion,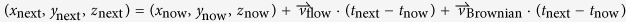. Based on the Navier-Stokes equation with lubrication approximation^20^, the non-dimensional capillary flow can be expressed as an analytical solution with radial and vertical components, 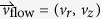 (see Supplementary Information)^7^,^8^,^20^. Note that particles can be considered following the velocities of capillary flow with nearly equal amplitude and phase since the Stokes response time^21^ is much shorter than the time step of our simulation (see Supplementary Information). For the Brownian motion, 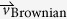 was generated according to a normal distributed probability density in the 3D domain (see Supplementary Information).

During the movement, the particles may become static and assembled with 3D hexagonal close packing when sinking to the bottom, 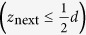 or near the droplet edge 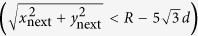 (*Status* = *Static*, Supplementary Fig. S2). To model the assembly of 3D hexagonal close packing of particles (Fig. 1b, the upper left corner), we pre-defined the locations of empty spots with 3D hexagonal close packing filling up the entire 3D domain. Along the particle’s moving direction, it first filled the outermost spot of the bottom layer, e.g. the spot in layer 1 and ring 1 (Supplementary Fig. S3). Subsequently, the upcoming particles in the same direction were successively filled into the adjacent empty spots with a priority order following the numerical sequence shown in Supplementary Fig. S3. The number of layers available for accommodating the particles is subject to the height of the local air-water interface. Ultimately, a 3D structure with hexagonal close packing of particles was formed in the inner part of the substrate, or in the wedge space along the contact line, leaving an ring-shaped pattern.

Alternatively, the particles may be adsorbed to the descending air-water interface when reaching the domain boundary, 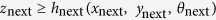, by which dimers would induce the cluster growing as a particle monolayer by recruiting monomers (Fig. 1b, the upper right corner). The dimers would float at the air-water interface once being adsorbed (*Status* = *Interface*, Supplementary Fig. S2)^4^, after that the dimers were considered as the smallest cluster. An effective attraction range (*R*_eff_ = 5*d*) was defined as a physical distance radiating from each particle belonging to an existing cluster. The effective attraction range was estimated based on the previous studies concerning particle-particle attraction between non-spherical particles^12^,^22^. Once a monomer collided the air-water interface, 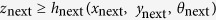, if it fell into the effective attraction range of an existing cluster, the monomer would be attracted and become part of the cluster. To model it, the location of each particle at air-water interface, including monomers at air-water interface and particles belonging to clusters, were first discretized and mapped into a fixed 2D grid (length × width = 1 μm × 1 μm, one particle per grid cell) projected from the air-water interface on the horizontal plane (Supplementary Fig. S4a,b). For each floating monomer, we first searched all grid cells within the radial range of *R*_eff_, and the grid cells occupied by a particle of a cluster close to the monomer were identified (Supplementary Fig. S4c,d). Next, based on the original coordinates of those identified particles in the 3D continuous domain, the closest one was identified as the “nearest particle” (Supplementary Fig. S4e). Afterward, a second search was conducted around the nearest particle to identify the adjacent particles with a distance of 1*d*, among which the one closest to the monomer was defined as the “second nearest particle” (Supplementary Fig. S4f). Thus, the floating monomer would move to the location between the nearest and the second nearest particle of that cluster (Supplementary Fig. S4g) and became part of the cluster with hexagonal close packing (*Status* = *Interface*, Supplementary Fig. S2).

In addition, we also modeled the cluster combination by including a factor of randomness (Fig. 1b, the lower left corner). At air-water interface, each cluster moved following the surface fluid flow as a whole (*Status* = *Mobile*, Supplementary Fig. S2), i.e. cluster can retain its own structure and the move with the average velocity of all member particles, i.e. (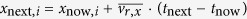, 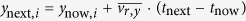, 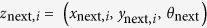, where *i* 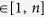, *n* = cluster particle number, 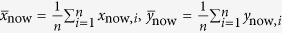, 
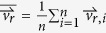
, 
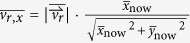
, 
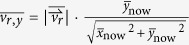
). Notably, clusters do not overlap with each other. Moreover, to embody the stochastic attribute, we introduced a random motion of clusters. Specifically, after moving with the surface flow, each cluster was allowed to perform a virtual walk in a randomly selected direction within the flattened air-water interface. During the virtual walk with *R*_eff_ as the maximum travel distance, the walking cluster would stop and combine with the clusters of first contact (which can be multiple), or return to its initial position if no cluster-cluster contact occurred.

Finally, the clusters may deform and accumulate along the droplet edge (Fig. 1b, lower right corner). Once the cluster approached to the droplet edge, 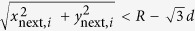 (*i* ∈ [1, *n*], *n* = cluster particle number), or encountered other immobile clusters, large cluster became immobilized and retains its original shape (*Status* = *Immobile*, Supplementary Fig. S2). In contrast, if the size of the cluster was less than a threshold (the total number of microspheres, *n*_max_ < 3,000, which is subject to the parametric study), the cluster would deform and became a particle monolayer with hexagonal close packing above the 3D ring structure. Similar to the formation of 3D hexagonal close packing of particles, the incoming cluster was broken into individual particles, which were successively filled into the empty spots near the existing particle monolayer with a priority order following the numerical sequence shown in Supplementary Fig. S5.

## Additional Information

**How to cite this article**: Xu, T. *et al*. Discrete Element Model for Suppression of Coffee-Ring Effect. *Sci. Rep.* **7**, 42817; doi: 10.1038/srep42817 (2017).

**Publisher's note:** Springer Nature remains neutral with regard to jurisdictional claims in published maps and institutional affiliations.

## Supplementary Material

Supplementary Information
