# Supplementary material for: Discrete Element Model for Suppression of Coffee-Ring Effect
Source: Sci Rep. 2017 Feb 20;7:42817. doi: 10.1038/srep42817 (PMC5316993; doi:10.1038/srep42817)
Supplement: Supplementary Information [file srep42817-s1.pdf]

Supplementary Information

for

Discrete Element Model for Suppression of Coffee-Ring Effect

Ting Xu,[1] Miu Ling Lam,[2,3,4] and Ting-Hsuan Chen[1,2,3,4]

[1]Department of Mechanical and Biomedical Engineering, City University of Hong Kong, 83 Tat Chee Avenue, Hong Kong Special Administrative Region

[2]School of Creative Media, City University of Hong Kong, 83 Tat Chee Avenue, Hong Kong Special Administrative Region

[3]Centre for Robotics and Automation, City University of Hong Kong, 83 Tat Chee Avenue, Hong Kong Special Administrative Region

[4]CityU Shenzhen Research Institute, 8 Yuexing 1st Road, Shenzhen Hi-Tech Industrial Park, Nanshan District, Shenzhen 518057, China

Correspondence and requests for materials should be addressed to M.L.L. (email: miu.lam@cityu.edu.hk) and T.-H.C. (email: thchen@cityu.edu.hk)

Spherical Cap of the 3D domain

The droplet shape was treated as a spherical cap to calculate the change of contact angle during evaporation.[1] This spherical cap assumption is valid with small value of Bond number (Bo), $\rho g R h_0/\sigma$, and capillary number (Ca), $\mu\overline{u_r}/\sigma$, which is the ratio of gravitational forces and viscosity to surface tension, respectively. Using the parameters of our simulation, in which ρ is the density of water (1000 kg/m^3), g is the gravity (9.8 m/s^2), R is the contact line radius of the droplet (1 mm), h_0 is the initial droplet (0.364 mm), σ is the surface tension coefficient (72 mN/m), μ is water viscosity (8.9 $\times 10^{-4}$ kg/s·m), and $\overline{u_r}$ is the average radial velocity of capillary flow (1 μm/s, see below), it yields Bo = 0.05 and Ca = 1.2 $\times 10^{-8}$. Thus, the assumption of spherical cap is valid throughout the process.

Capillary Flow

The velocity field of the capillary flow was based on the analytical solutions by Hu et al.[1,2] Using cylindrical coordinate, the dimensionless flow velocity in radial axis, $\widetilde{v_r}$, and z-axis, $\widetilde{v_z}$, were shown in follows:

$$\widetilde{v_r}, = \frac{3}{8}\frac{1}{1-\tilde{t}}\frac{1}{\tilde{r}}\left[(1-\tilde{r}^2) - (1-\tilde{r}^2)^{-\lambda}\right]\left(\frac{\tilde{z}^2}{\tilde{h}^2} - 2\frac{\tilde{z}}{\tilde{h}}\right) + \frac{\tilde{r}\,{h_0}^2\tilde{h}}{R^2}\left[\tilde{J}\lambda(1-\tilde{r}^2)^{-\lambda-1}+1\right]\left(\frac{\tilde{z}}{\tilde{h}} - \frac{3}{2}\frac{\tilde{z}^2}{\tilde{h}^2}\right) + \frac{Mah_0\tilde{h}}{4R}\frac{\mathrm{d}\tilde{T}}{\mathrm{d}\tilde{r}}\left(2\frac{\tilde{z}}{\tilde{h}} - 3\frac{\tilde{z}^2}{\tilde{h}^2}\right)$$

$$\widetilde{v_z}, = \frac{3}{4}\frac{1}{1-\tilde{t}}\left[\lambda(1-\tilde{r}^2)^{-\lambda-1}+1)\right]\left(\frac{\tilde{z}^3}{3\tilde{h}^2} - \frac{\tilde{z}^2}{\tilde{h}}\right) + \frac{3}{2}\frac{1}{1-\tilde{t}}\left[(1-\tilde{r}^2) - (1-\tilde{r}^2)^{-\lambda}\right]\left(\frac{\tilde{z}^2}{2\tilde{h}^2} - \frac{\tilde{z}^3}{3\tilde{h}^3}\right)\left(-\frac{h_0}{t_f}\right) - \frac{{h_0}^2}{R^2}\left(\tilde{J}\lambda(1-\tilde{r}^2)^{-\lambda-1}+1\right)\left(\tilde{z}^2 - \frac{\tilde{z}^3}{\tilde{h}}\right) - \frac{\tilde{r}^2\,{h_0}^2}{R^2}\tilde{J}\lambda(\lambda+1)(1-\tilde{r}^2)^{-\lambda-2}\left(\tilde{z}^2 - \frac{\tilde{z}^3}{\tilde{h}}\right) - \frac{\tilde{r}\,{h_0}^2}{R^2}\left(\tilde{J}\lambda(1-\tilde{r}^2)^{-\lambda-1}+1\right)\left(\frac{\tilde{z}^3}{\tilde{h}^2}\right)\frac{\partial\tilde{h}}{\partial\tilde{r}} - \frac{Mah_0}{4R}\left(\tilde{z}^2 - \frac{\tilde{z}^3}{\tilde{h}}\right)\left(\frac{d^2\tilde{T}}{d\tilde{r}^2} + \frac{1}{\tilde{r}}\frac{d\tilde{T}}{d\tilde{r}}\right) - \frac{Mah_0}{4R}\left(\frac{\tilde{z}^3}{\tilde{h}^2}\right)\frac{\mathrm{d}\tilde{T}}{\mathrm{d}\tilde{r}}\frac{\partial\tilde{h}}{\partial\tilde{r}}$$

where the dimensionless variables are:

$$\widetilde{v_r} = \frac{v_r t_\mathrm{f}}{R},\ \widetilde{v_z} = \frac{v_z t_\mathrm{f}}{h_0},\ \tilde{t} = \frac{t}{t_\mathrm{f}},\ \tilde{z} = \frac{z}{h_0},\ \tilde{r} = \frac{r}{R},\ \tilde{h} = \frac{h}{h_0},\ \dot{h} = \frac{h_0}{t_\mathrm{f}},\ \tilde{J} = \frac{J_0(\theta)}{\rho\dot{h}},\ \tilde{T} = \frac{T-T_\mathrm{c}}{T_\mathrm{e}-T_\mathrm{c}},$$

and h_0 denotes the initial droplet height (0.364 mm), R is the contact line radius (1 mm), ρ is the density of water (1000 kg/m^3), θ is the contact angle (40° when $t = 0$), $\lambda = \frac{1}{2} - \frac{\theta}{\pi}$ is the parameter reflecting the uniformity of evaporative flux, and Ma is the Marangoni number (8, experimental approximated).[2] $J_0(\theta)$ is the dimensionless evaporative flux at the top of the droplet surface, which can be denoted as[2,3]

$$J_0(\theta) = \frac{Dc_v(1-H)}{R}(0.27\theta^2 + 1.30)\left[0.6381 - 0.2239\left(\theta - \frac{\pi}{4}\right)^2\right]$$

where D is the vapor diffusivity (26.1 mm^2/s at 25°C), c_v is the saturated vapor concentration on the droplet surface (2.32×10^{-8} g/mm^3), and H indicates the relative humidity of the ambient air (0.38).

To calculate the total drying time t_f, two evaporation phases were conducted. In phase 1, the contact line was pinned while the droplet height, h, continuously decayed in the vertical direction. The h and the contact angle θ were updated every second based on the approximate evaporation rate, $\dot{m} = -\pi RD(1-H)c_v(0.27\theta^2 + 1.3)$,[1] in which R is the contact line radius (1 mm), D is the vapor diffusivity (26.1 mm^2/s at 25°C), c_v is the saturated vapor concentration on the droplet surface 2.32×10^{-8} g/mm^3), and H indicates the relative humidity of the ambient air (0.38). The phase 2 of evaporation started when θ became 4°, in which θ was kept at 4° while the contact line gradually receded.[1,4] Afterward, the evaporation was completed when the contact line radius shrunk to zero. Thus, the duration combining phase 1 and 2 was then used as the total drying time t_f. Based on our settings, the duration for phase 1 is 341s, and t_f is 378 s.

The temperature T of air-water interface was calculated based on the following equation:[2]

$$T - T_\infty = h_0 H_v J(\tilde{r}, \theta)\sqrt{1 + \left(\frac{dh}{dr}\right)^2}\left(\frac{\tilde{h}}{k_1} + \frac{\tilde{h}_g}{k_2}\right)$$

where $T_\infty = 25$ °C is the ambient temperature, H_v is the latent heat of evaporation of water (-2.26 $\times 10^3$ kJ/kg), k_1 is the thermal conductivity of the water (0.6 W/m·K), k_2 is the thermal

conductivity of the substrate (1.1 W/m·K), $\widetilde{h_g} = \frac{h_g}{h_0}$ is the dimensionless substrate thickness (h_g = 0.5 mm). Based on this equation, T_e and T_c are the surface temperature at the edge ($\tilde{r} =$ 0.999) and at the top of the droplet ($\tilde{r} = 0$).

Brownian Motion

The vector of Brownian motion, $\vec{v}_{\mathrm{Brownian}}$, was generated according to a normal distributed probability density in the 3D domain,

$$f(\mathbf{X}, \mathbf{\Sigma}) = \frac{\exp\left(-\frac{1}{2}(\mathbf{X})^{\mathrm{T}}\mathbf{\Sigma}^{-1}(\mathbf{X})\right)}{\sqrt{(2\pi)^3|\mathbf{\Sigma}|}}$$

where **X** is a coordinate vector of the 3D domain (*x*, *y*, *z*), $\mathbf{\Sigma} = \begin{bmatrix} \frac{k_B T t}{6\pi\mu r} & 0 & 0 \\ 0 & \frac{k_B T t}{6\pi\mu r} & 0 \\ 0 & 0 & \frac{k_B T t}{6\pi\mu r} \end{bmatrix}$ is the covariance matrix, *T* is temperature (298.15 K, which is equal to 25°C), k_B is Boltzmann's constant (1.38 × 10^{-23} J/K), μ is water viscosity (8.9 ×10^{-4} kg/s·m), *r* is particle radius (0.5 µm), *t* is time step (1 s), and $|\mathbf{\Sigma}|$ is the determinant of $\mathbf{\Sigma}$.

Stokes Response Time

To study the influence of capillary flow velocity when the particle is present, we analyzed the Stokes response time,[5] $\tau_s = d^2\rho_p/(18\mu)$, where *d* is the particle diameter, ρ_p is the density of particle, and μ is fluid dynamic viscosity. Using the parameter settings of our simulation, *d* = 1 µm, ρ_p = 1050 kg/m^3, and μ = 8.9 × 10^{-4} kg/m·s, the Stokes response time was calculated as 6.55 × 10^{-8} s, which is significantly shorter than the time step in our simulation ($t_{\mathrm{next}} - t_{\mathrm{now}}$ = 1 s). Thus, the particles can be considered following the velocities of capillary flow with nearly equal amplitude and phase.

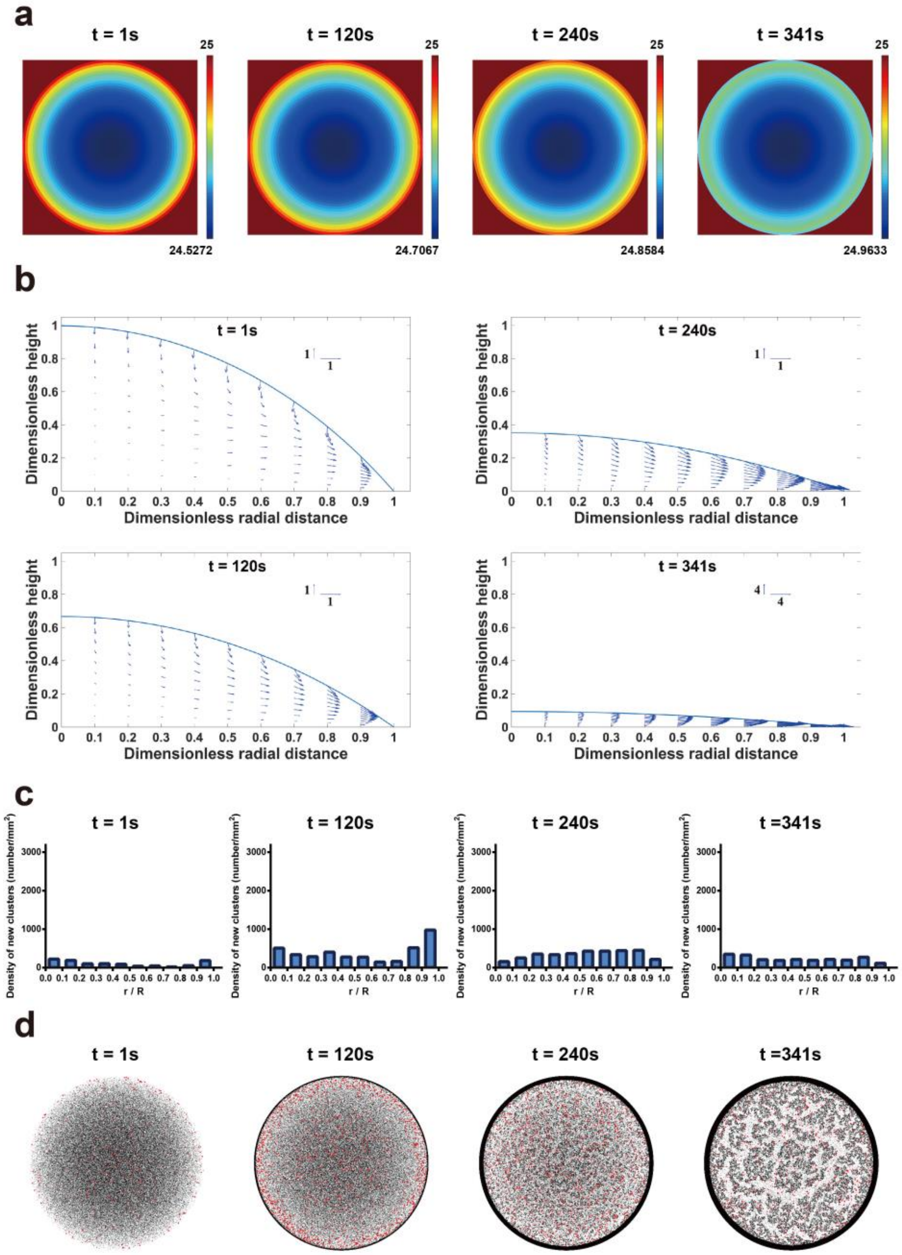

Figure S1 | Reduced circulatory flow based on increased thermal conductivity of the substrate (100 W/m·K) while the Marangoni number remained unchanged (Ma = 8). (**a**) The color map of temperature distribution during evaporation. (**b**) The velocity field of the circulatory flow at the side view. (**c**) The histogram of the location of newly adsorbed dimers during evaporation based on total 1,500,000 microspheres with 66.7% dimers (*w*/*w*). (**d**) The top view of newly adsorbed dimers (labeled red) at different time based on total 1,500,000 microspheres with 66.7% dimers (*w*/*w*).

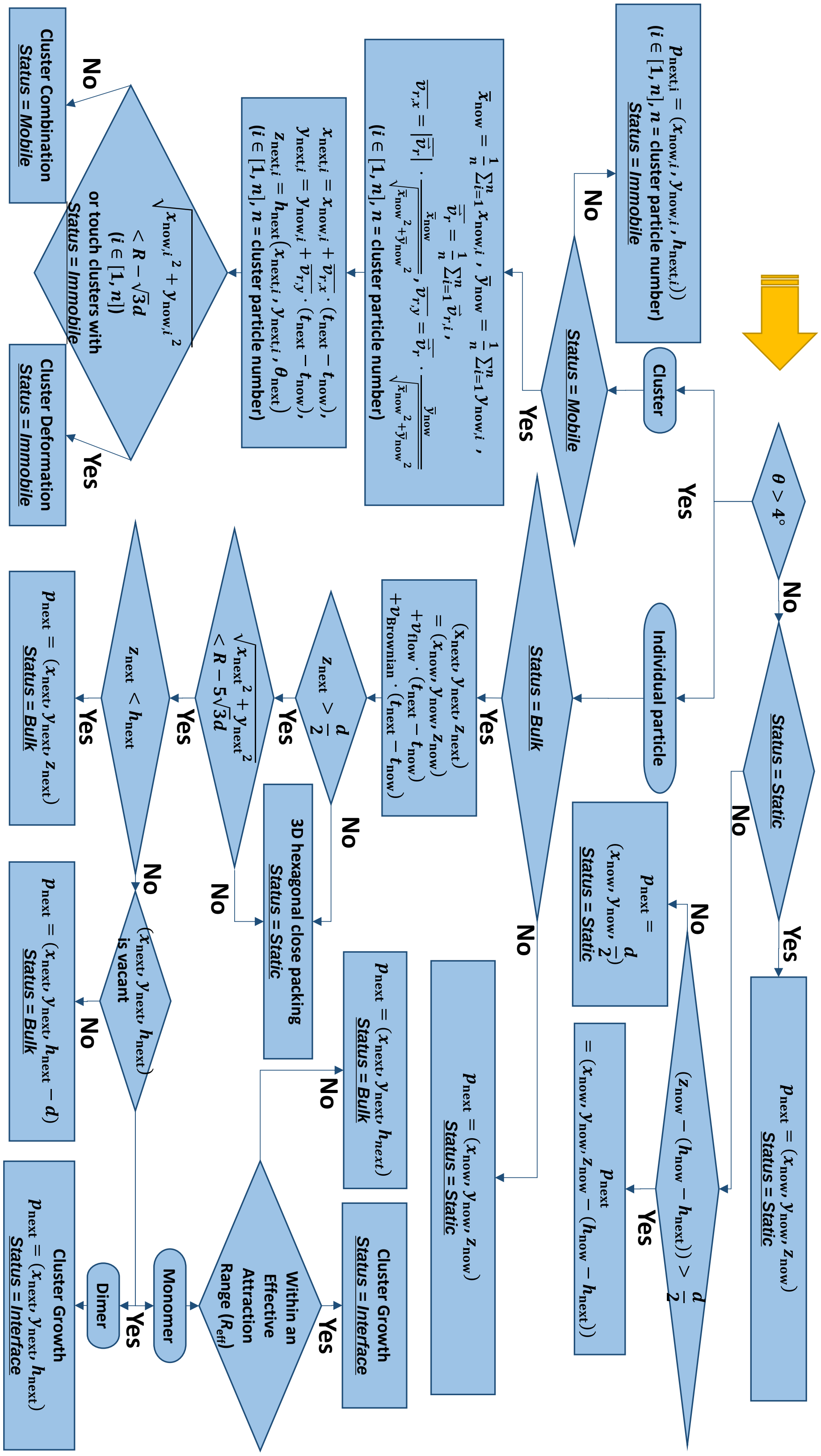

Figure S2 | The flow chart showing a systematic framework to determine the status of each particle so an appropriate particle motion can be applied.

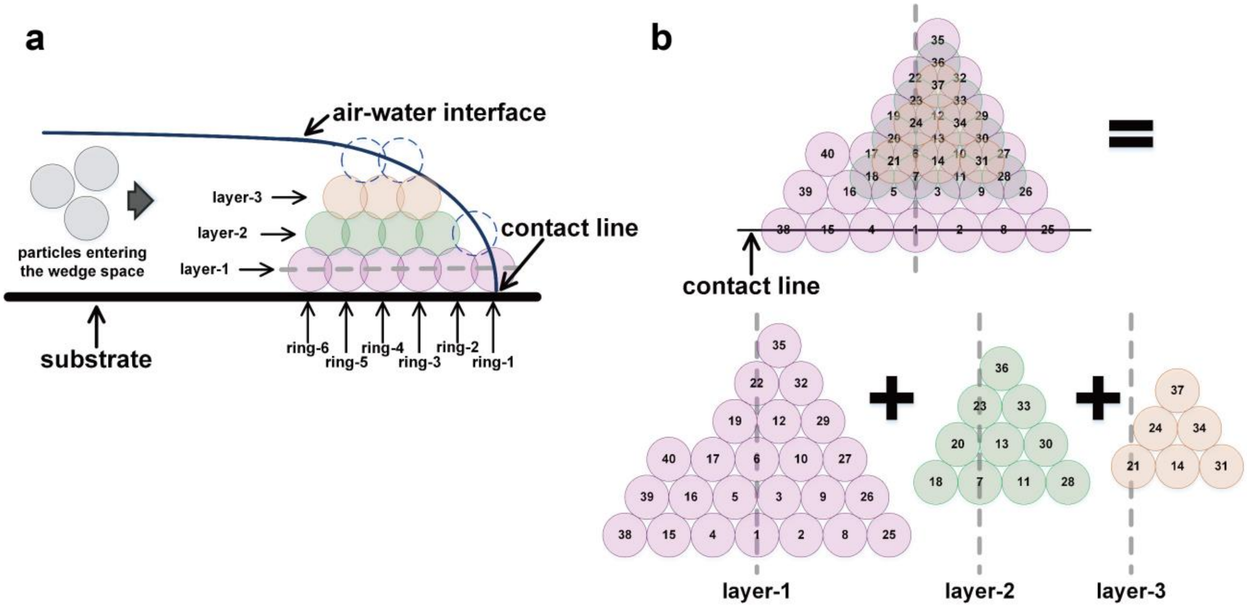

Figure S3 | The schematic of the 3D hexagonal close packing of particles in the bulk solution. (**a**) The side view of the pre-defined empty spots in number of rings and layers with 3D hexagonal close packing. (**b**) The top view showing the particles being filled in the empty spots of the 3D hexagonal close packing, where the numbers in particles indicates the sequence of assembly.

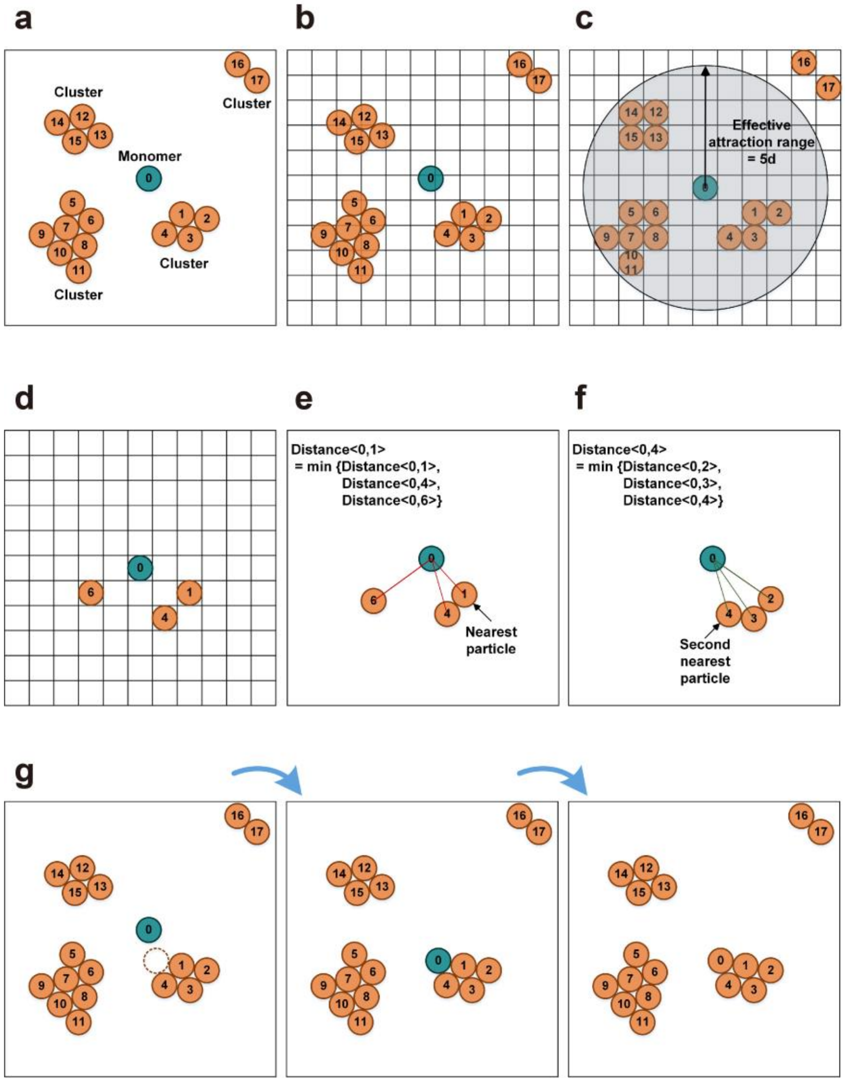

Figure S4 | The schematic of cluster growth. (**a**) A monomer surrounding by multiple clusters in the continuous domain. (**b**) The location of each particle at air-water interface, including monomers and particles belonging clusters, were discretized and mapped into a fixed 2D grid (length × width = 1 μm × 1 μm, one particle per grid cell). (**c**) Using the grid cell occupied by the monomer as the center, we searched all grid cells within the radial range of $R_{\mathrm{eff}} = 5d$. (**d**) The grid cells occupied by a particle of a cluster close to the monomer were identified. (**e**) Based on the original coordinates of those identified particles, the closest one was identified as the "nearest particle". (**f**) A second search was conducted around the nearest particle to identify the adjacent particles with a distance of $1d$, among which the one closest to the original monomer was defined as the "second nearest particle". (**g**) The monomer was move to the location between the nearest and the second nearest particle of that cluster with hexagonal close packing.

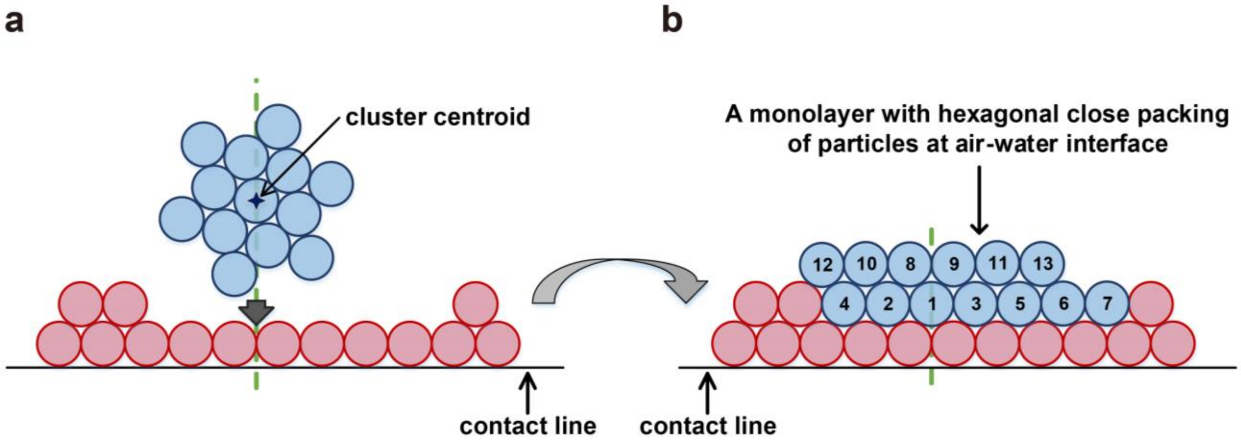

Figure S5 | The schematic of cluster deformation. (**a**) The top view showing an incoming cluster towards the particle monolayer with hexagonal close packing. (**b**) The cluster was broken into individual particles, which would be successively filled into empty spots near the existing particle monolayer, where the numbers in particles indicates the sequence of assembly.

.

References

1 Hu, H. & Larson, R. G. Evaporation of a sessile droplet on a substrate. *J. Phys. Chem. B* **106**, 1334-1344 (2002).

2 Hu, H. & Larson, R. G. Marangoni effect reverses coffee-ring depositions. *J. Phys. Chem. B* **110**, 7090-7094 (2006).

3 Hu, H. & Larson, R. G. Analysis of the microfluid flow in an evaporating sessile droplet. *Langmuir* **21**, 3963-3971 (2005).

4 Yunker, P. J., Still, T., Lohr, M. A. & Yodh, A. G. Suppression of the coffee-ring effect by shape-dependent capillary interactions. *Nature* **476**, 308-311 (2011).

5 Smith, S. J. & Friedrichs, C. T. Image processing methods for in situ estimation of cohesive sediment floc size, settling velocity, and density. *Limnol. Oceanogr. Methods* **13**, 250-264 (2015).
